# Comparative analysis of COVID-19 and influenza prevalence among Egyptian pilgrims returning from Hajj and Umrah in 2022: epidemiology, clinical characteristics, and genomic sequencing

**DOI:** 10.1186/s13690-023-01229-6

**Published:** 2024-01-12

**Authors:** Amr Kandeel, Manal Fahim, Ola Deghedy, Hala BahaaEldin, Wael H. Roshdy, Mohamed Kamal Khalifa, Ahmed Kandeil, Rabeh El Shesheny, Amel Naguib, Mohamad AbdelFatah, Salma Afifi, Khaled Abdel Ghaffar

**Affiliations:** 1 Preventive Sector, Ministry of Health and Population, Governmental District, New Administrative Capital, Cairo, Egypt; 2 Department of Epidemiology and Surveillance, Preventive Sector, Ministry of Health and Population, Governmental District, New Administrative Capital, Cairo, Egypt; 3 Central Public Health Laboratory, Ministry of Health and Population, Governmental District, New Administrative Capital, Cairo, Egypt; 4 Centre of Scientific Excellence for Influenza Viruses, National Research Centre, 12622 Dokki, Giza, Egypt; 5 Ministry of Health and Population Consultant, Governmental District, New Administrative Capital, Cairo, Egypt; 6 Ministry of Health and Population, Governmental District, New Administrative Capital, Cairo, Egypt

**Keywords:** Influenza A, Influenza B, SARS-CoV-2, COVID-19, Breakthrough Infection, Vaccine effectiveness

## Abstract

**Purpose:**

To describe the changes that occurred in the SARS-CoV-2 and influenza Prevalence, epidemiology, clinical picture, and prevalent genotypes among the Egyptian pilgrims returning from Hajj and Umrah 2022 seasons.

**Methods:**

Pilgrims were contacted at the airport and invited to participate in the survey. Pilgrims who consented were interviewed using a standardized line list that included participant demographics, respiratory symptoms if any, previous COVID-19 infection, influenza vaccination whereas COVID-19 vaccination information were collected from vaccination cards. Participants were asked to provide throat and nasopharyngeal swabs for SARS-CoV-2 and influenza testing using RT-PCR and a subset of isolates were sequenced. Descriptive data analysis was performed to describe the epidemiology and clinical symptoms of SARS-CoV-2 and influenza. Prevalence rates of SARS-CoV-2 and influenza during Hajj were calculated and compared to Umrah surveys using chi^2^ and t-test with a significance level < 0.05.

**Results:**

Overall, 3,862 Egyptian pilgrims enrolled, their mean age was 50.5 ± 47 years, half of them were > 50 years of age and 58.2% were males. Of them, 384 (9.9%) tested positive for SARS-CoV-2 and 51 (1.3%) for influenza viruses. Prevalence of SARS-CoV-2 infections (vaccine breakthrough) increased significantly between the Umrah and Hajj surveys (6.7% vs. 9.9%, *p* < 0.001), and variants of the virus varied considerably. Whereas no significant difference was found in influenza prevalence, vaccine coverage and vaccine breakthrough infection rates (11.7 vs. 9.2%, 26.9 vs. 26.8%, and 1.4 vs. 1.1% respectively).

**Conclusions:**

SARS-CoV-2 prevalence among Egyptian pilgrims returning from Hajj in July increased with reduced vaccine effectiveness compared to Umrah in March 2022 suggesting a possible wave of SARS-CoV-2 in the upcoming winter.

## Introduction

Hajj is a pilgrimage that millions of Muslims from around the world perform every year during the holy month of Dhu’al-Hijjah [1]. Typically, over two million Muslims gather in the holy cities of Mecca and Medina to perform Hajj every year. Due to the Coronavirus disease 2019 (COVID-19) pandemic, Hajj was suspended in the 2020–2021 seasons, and only one million Muslims were able to make the Hajj in 2022 [2]. A special set of rules has been implemented for Hajj pilgrims during the 2022 Hajj to prevent COVID-19 from spreading. The event was restricted to people under 65 years old who have been vaccinated against COVID-19 and submitted a negative test result within 72 h of leaving for the kingdom. In addition, a strict physical distancing was also enforced by the Saudi Ministry of Health during the pilgrims’ transition between the two Holy Mosques and the central areas of Makkah and Madinah [3]. Saudi health authorities allowed Hajj pilgrimage after 2022 season without COVID-19 restrictions, maximum number of pilgrims, or upper age limits, and kept requirement of vaccination with COVID-19, influenza, and ACYW meningitis vaccines [4]. According to the Saudi General Authority for Statistics, 2022 Hajj was performed between 17 and 23 July by 899,353 pilgrims, including 779,919 from outside of the Kingdom [2]. The outsiders consisted of 21.4% Arabs, 53.8% Asians, 13.2% Africans, and 11.6% Europeans, Americans, and Australians [2].

Every year, approximately 80,000 Egyptians perform the Hajj, while 13,700 pilgrims performed the Hajj in 2022, with 80% arriving at Cairo airport and 20% at Borg-El Arab airport. Pilgrims normally stay during the Hajj for a few days to a few weeks. The Egyptian health authorities requested that all pilgrims be vaccinated against COVID-19 and influenza before leaving Egypt to prevent the importation of acute respiratory infections. .

Respiratory symptoms are common among Hajj pilgrims with cough being the most common symptom [5]. Acute respiratory infections (ARI) are the most common diseases among pilgrims and a major cause for hospital and intensive care unit admission during the Hajj [6]. The most common causes of ARI are the respiratory viruses especially rhinovirus, influenza, and coronaviruses followed by bacteria, including *Streptococcus pneumonia, Hemophilus influenza, Staphylococcus aureus and Klebsiella pneumonia* [5]. Risk factors could include crowded conditions and close contact among pilgrims, as well as the climatic conditions and air pollution arround the holy sites [7].

The Egyptian Ministry of Health and Population (MoHP) conducts a survey of pilgrims returning from Hajj to monitor influenza activity and assess the risk of imported acute respiratory viruses every year since 2009 [8]. To guide preventive and control measures during the 2022 Hajj, a survey of Egyptian pilgrims returning from Ramadan Umrah -the second largest Islamic pilgrimage- was conducted between 30th April and 5th May 2022 [9]. This report aims at estimating the prevalence of COVID-19 and influenza among Egyptian pilgrims returning from the Hajj, as well as their epidemiology, clinical picture, severity, and predominant genotypes. The study results was compared with Ramadan Umrah survey results to examine the changes in prevalence and genotypes between May and July 2022 [9].

### Research question

Are there any changes in the prevalence and essential aspects of COVID-19 and influenza virus infections among Egyptian pilgrims returning from two mass gatherings in Saudi Arabia three months apart?

### Methods

We used the same methods for Ramadan Umrah surveys with the exception that we inflated the sample size to cover 25% of pilgrims or approximately 3,500 pilgrims, of which 700 returning through Borg Elarab Airport, and 2,800 returned through Cairo Airport. We investigated a larger sample size in order to detect and treat the COVID-19 and influenza patients, while taking into account laboratory and epidemiological capabilities [9]. Of the Egyptian pilgrims who attended the Hajj 2022 mass gathering in Saudi Arabia, or about 3,500. Additionally, the second most important airport used by returning pilgrims was involved in Hajj survey to provide a more accurate assessment of SARS-CoV-2 and influenza risks on the Egyptian population [9].

### Study design

A cross-sectional study was conducted among Egyptian pilgrims who returned from Hajj 2022 between July 12 and 20, 2022.

### Setting

The study was conducted in the two airports serving the Hajj and Umrah pilgrims in Egypt. Cairo airport is the main airport in Cairo, the capital, where 80% of pilgrims arrive, while Borg Elarab is the second airport in the north of Egypt, where 20% arrive.

### Participants

Pilgrims returning from the Hajj were contacted while on their way to the passport clearance services and invited to participate voluntarily in the survey. Survey teams were instructed to choose pilgrims throughout the area without regard to age, sex, or illness status. Only pilgrims who verbally consented were eligible to participate in the survey, while those who did not consent were excluded. Participants were briefed on the study objectives and methods, then interviewed face-to-face using a standardized line list. The interview was conducted by MoHP epidemiologists at the airport clinic to protect participants’ privacy and confidentiality.

### Data collection tool

We used a line list to collect participants’ demographic data including age, gender, residence, acute respiratory symptoms in the last ten days, influenza vaccination status, and previous COVID-19 infections. COVID-19 vaccination information including vaccination status, date of the last dose received, and type were collected from the vaccination cards, if available, or from the study participants if they were unable to provide the cards during the interview. Data was entered into an excel database, SARS-CoV-2 and influenza virus-positive patients were contacted for follow-up, medical advice, and referral for healthcare if needed.

### Defined variables

A vaccine breakthrough infection (VBTI) was defined as an infection with SARS-CoV-2 or influenza confirmed by RT-PCR in a fully vaccinated individual. Because pre-departure COVID-19 vaccination was mandatory for all pilgrims, COVID-19 prevalence rate was the same as the VBTI. Subjects with previous COVID-19 infection were considered to have hybrid immunity.

### Laboratory procedures

Consented participants were asked to provide nasopharyngeal and oropharyngeal (NP/OP) swabs collected with flocked swabs and stored in viral transport media (VTM), preserved in nitrogen tanks, and transported to the Central Public Health Laboratory (CPHL) in Cairo within 24 h for influenza and SARS-CoV-2 testing by Real-Time polymerase chain reaction (RT-PCR). Assigned Lab technician is responsible for samples collection at the airport; samples transported to the lab at the same day. CPHL working 24/7; once samples arrive to CPHL (within maximum 2 h) samples registered and barcoded and lab testing starts immediately.

### Detection of Influenza and SARS-CoV-2 viruses

The nucleic acid was extracted from clinical specimens using chemagic 360 equipment (PerkinElmer Inc). A RT-PCR influenza and SARS-CoV-2 screening test was conducted on all specimens [10]. Specimens that tested positive for influenza A were further tested for A subtype according to WHO guidelines [11]. A cobas ® SARS-CoV-2 RT-PCR qualitative nucleic acid test Kit was used to identify SARS-CoV-2 RNA (ORF1ab and E genes) (Cobas 6800 system/Roche Diagnostics) [12].

### SARS-CoV-2 whole-genome sequencing

Nucleic acid was freshly extracted from all the original selected specimens for whole-genome sequencing. According to the manufacturer’s instructions, all specimens were processed using the Illumina COVIDSeq protocol (Illumina, San Diego, CA). Using random hexamer primers, the first-strand synthesis was followed by two multiplex PCR reactions. Using the 2.0 fluorometer (Invitrogen Inc., Waltham, MA), pooled samples were quantified, then normalized to 4 nM concentration for sequencing on the Illumina MiSeq platform (Illumina Inc, San Diego, CA, USA).

A sequence analysis technique available with the CovidSeq assay is genotyping and strain typing of SARS-CoV-2 variants, which requires depth coverage of at least 100 strain-distinguishing regions of ORF1a (open reading frame 1a), ORFs, Ns, and ORF8 genes to achieve adequate sequencing. There was no mix of virus populations in any of the samples, and all of them produced strain-typeable sequences. In CLC Genomics Workbench version 20 (CLC Bio, Qiagen), reads were aligned with the reference genome (NC_045512.2). For the detected mutations in Hajj samples, enrichment analysis was conducted in comparison with 113 random samples from the circulating SARS-Cov2 variants obtained from Egyptian patients presenting to the national Egyptian surveillance for SARS-CoV-2 during the same period. Pangolin (https://pangolin.cog-uk.io/) criteria were used to classify the lineage and clade of the collected samples.

### Data analysis

Prevalence of COVID-19 and influenza was calculated by dividing the number of patients who tested positive for the specific virus by the number of study participants. Rates were weighted using the flight number and date of arrival. Descriptive data analysis was performed using SPSS ver. 25 for the demographic and epidemiologic characteristics of patients with COVID-19 and influenza viral infection using numbers and frequencies. SARS-CoV-2 positive and negative patients were compared using the chi^2^ and t-test, with a significance level of < 0.05 to estimate the effectiveness of different types of vaccines, booster doses, and different vaccination regimens for the prevention of SARS-CoV-2 infection. Prevalence of COVID-19 was stratified according to the time since the last dose of vaccination in order to investigate the duration of vaccine effectiveness.

## Results

### Demographic, epidemiologic, and clinical characteristics of the study subjects

Overall, 3,862 Egyptian pilgrims were enrolled between 12 and 20 July 2022 including 3,220 (83.4%) at Cairo airport and 642 (16.6%) at Borg Elarab airport. The mean age of the study participants was 50.5 ± 47, with almost half of them older than 50 years of age and 58.2% males. Of them, 165 (4.3%) reported having acute respiratory (AR) symptoms ten days prior to arrival to Egypt, and 100 (2.6%) contacted patients with AR symptoms. Of all study participants, 384 (9.9%) tested positive for SARs-CoV-2, 51 (1.3%) for influenza viruses, and one positive for both viruses (Table 1).

**Table 1:** Characteristics of study subjects and patients by viral cause of acute respiratory infection among the Egyptian pilgrims returning from 2022 Hajj

	All subjects (n = 3,862)		Positive for SARS-CoV-2 (n = 384)		Positive for influenza viruses (n = 51)		Negative for both viruses (n = 3,426)*		P value
Arrival date	No.	%	No.	%	No.	%	No.	%	
12 July	126	3.3	31	24.6	2	1.6	93	73.8	
13 July	290	7.5	67	23.1	3	1.0	220	75.7	< 0.001
14 July	1050	27.2	121	11.5	6	0.6	923	87.9	
15 July	589	15.3	51	8.7	8	1.4	530	90.0	
16 July	541	14.0	49	9.1	0	0.0	492	90.9	
17 July	412	10.7	26	6.3	11	2.7	375	91.0	
18 July	310	8.0	16	5.2	8	2.6	286	92.3	
19 July	404	10.4	16	4.0	10	2.5	377	93.6	
20 July	140	3.6	7	5.0	3	2.1	130	92.9	
Arrival airport									
Cairo	3,220	83.4	323	10.0	44	1.4	2,852	88.6	0.778
Borg Elarab	642	16.6	61	9.5	7	1.1	574	89.4	
Mean age years	50.5 ± 47		49.9 ± 10		49.1 ± 13		50.6 ± 50		0.491
Age groups									
1–14	7	0.2	1	14.3	0	0.0	6	85.7	0.323
15–35	366	9.5	36	9.8	6	1.6	324	88.6	
36–50	1451	37.6	143	9.8	22	1.6	1286	88.6	
51–65	1744	45.2	179	10.3	19	1.1	1546	88.6	
> 65	81	2.1	8	9.9	4	4.9	69	85.2	
Missing	212	5.5	17	8.0	0	0.0	195	92.0	
Gender									
Males	2,249	58.2	226	10.0	35	1.6	1,988	88.4	0.303
Females	1,612	41.8	158	9.8	16	1.0	1,438	89.2	
Region									
Lower Egypt	1,591	41.2	133	8.4	18	1.1	1,440	90.5	0.015
Urban governorates	1,247	32.2	148	11.9	12	1.0	1,087	87.2	
Upper Egypt	593	15.4	53	8.9	13	2.2	527	88.9	
Frontier	116	3.0	13	11.2	2	1.7	101	87.1	
Missing	314	8.1	37	11.8	6	1.9	271	86.3	
Symptoms									
Symptomatic within 10 days before arrival	165	4.3%	23	6.0%	8	15.7%	134	3.9%	< 0.001
Symptoms after arrival	20	0.5%	16	4.2%	4	7.8%	0	0.0%	
Unable to reach	153	4.0%	138	35.9%	15	29.4%	0	0.0%	
No symptoms reported	3524	91.2%	207	53.9%	24	47.1%	3292	96.1%	
Types of symptoms									
Fever	10	0.3	0	0.0	1	2.0	9	0.3	0.035
Cough	102	2.6	17	4.4	4	7.8	81	2.4	0.006
Rhinorrhea	71	1.8	6	1.6	3	5.9	62	1.8	0.091
Sore throat	40	1.0	3	0.8	2	3.9	35	1.0	0.111
Dyspnea	11	0.3	2	0.5	1	2.0	8	0.2	0.059
Contacted ARI case									
Yes	100	2.6	10	10.0	1	1.0	89	89.0	0.951
No	2,940	76.1	278	9.5	37	1.3	2,624	89.2	
Not sure	822	21.3	96	11.7	13	1.6	713	86.7	

*One patient had SARS-CoV-2/influenza coinfection was excluded from the comparison

During the first three days of the survey, the prevalence of SARS-CoV-2 infection was significantly higher (Range: 11.5 to 24.6%) compared to that over the last four days of the survey (Range: 4.0 to 9.1%). While influenza prevalence was higher in the last four days (Range: 2.1 to 2.7%) than in the first five days (Range: 0.0-1.6%) (Table 1).

Prevalence of SARS-CoV-2 infection was significantly higher among residents of Egypt’s urban and frontier governorates compared to residents of upper and lower Egypt regions (11.9 and 11.2% vs. 8.9 and 8.4% respectively), while the prevalence of influenza was higher among upper Egypt residents compared to the urban, lower Egypt and frontier governorates (2.2% vs. 1.0, 1.1 and 1.7% respectively, *p* < 0.05) (Table 1).

Influenza patients reported having acute respiratory symptoms prior to arrival to Egypt more than SARS-CoV-2 and negatively tested patients (15.7 vs. 6.0 and 3.9% respectively, *p* < 0.001). Among influenza-positive patients, 4 (7.8%) reported symptoms after arrival, including 2 (50.0%) sought medical advice. Among patients positive for SARS-CoV-2, 16 (4.2%) reported symptoms after arrival, including 3 (18.8%) sought medical advice, but none of them required hospitalization (Table 2).

**Table 2:** COVID-19 vaccination status of the Egyptian pilgrims returning from the 2022 hajj

COVID-19 vaccination	All subjects (n = 3,862)		Positive for SARS-CoV-2 (n = 385)		Negative for SARS-CoV-2 (n = 3,477)		P-value
	No.	%	No.	%	No.	%	
Vaccine type							
Viral vector	1,465	38.0	146	9.9	1,319	90.0	0.773
mRNA	492	12.7	46	9.3	446	90.7	
Inactivated	542	14.0	55	10.1	487	89.9	
Heterologous	843	21.8	92	10.9	751	89.1	
DK	520	13.5	46	8.8	474	91.2	
vaccine booster dose							
Yes	2,027	52.5	223	11.0	1,804	89.0	0.012
No	1,835	47.5	162	8.8	1,673	91.2	
Duration since the last dose							
One month	490	12.7	52	10.6	438	89.4	0.618
1–3 months	611	15.8	63	10.3	548	89.7	
> 3 months	1,687	43.7	156	9.2	1,531	90.8	
DK	1,074	27.8	114	10.6	960	89.4	
Previous COVID-19 infection							
Yes (Hybrid immunity)	395	10.2	44	11.1	351	88.9	0.575
No	2,699	69.9	261	9.7	2,438	90.3	
Not sure	768	19.9	80	10.4	688	89.6	

In comparison with SARS-CoV-2 infected patients, influenza patients had significantly higher rates of fever and cough (2.0, 7.7% vs. 0.0 and 4.4%, respectively), and insignificantly higher rates of rhinorrhea, sore throats, and dyspnea (5.9, 3.9, and 2.7% vs. 1.6, 0.8, and 0.5%, respectively) (Table 1). There was no significant difference found between pilgrims tested positive for SARS-CoV-2 or influenza and those who tested negative regarding the port of arrival, age groups, gender, and history of contact with a patient with AR symptoms (Table 1).

### Vaccination

#### SARS-CoV-2

Among all study participants, 38% were fully vaccinated with a viral vector vaccine type, 14% had inactivated, and 12.7% had an mRNA vaccine. Additionally, 21.8% had more than one-type schedule, 13.4% did not know the vaccine type they had, and 52.5% received booster doses of a COVID-19 vaccine (Table 2). The COVID-19 prevalence or vaccine breakthrough infection rate (VBTI) was 9.9% (385/3,862), no differences in the VBTI rate were found between different vaccine types or vaccine administration schedules (Table 2). There was also no significant difference between VBTI rates regarding duration since the last vaccine dose or the previous infection with COVID-19 (Hybrid immunity). Interestingly, the VBTI rate was higher among booster dose receivers than those who had not received booster doses (11.0 vs. 8.8%, *p* < 0.05) (Table 2).

#### Influenza

Among all study subjects, 1,039 (26.9%) mentioned receiving the pre-departure influenza vaccine. VBTI for influenza was 1.4% (Table 3).

**Table 3:** A comparison between characteristics of the viruses isolated from the Egyptian pilgrims returning from 2022 Hajj compared to Ramadan Umrah pilgrims

Viruses	Ramadan Umrah (n = 1,003)		Hajj (n = 3,862)		P value
	No.	Percent	No.	Percent	
Positive	76	7.6%	436	11.3%	< 0.001
SARS-CoV-2					
Incidence/Vaccine breakthrough rate	67	6.7%	384	9.9%	< 0.001
Genotypes					
Number sequenced	17	22.4	143	37.2	0.08
B.1.1.529	1	6.0%	6	4.2%	0.551
BA.1	2	11.8%	0	0.0%	NA
BA.2	10	58.8%	8	5.6%	< 0.001
BA.2.75	0	0.0%	1	0.7%	NA
BA.2.76	0	0.0%	2	1.4%	NA
BA.5	0	0.0%	1	0.7%	NA
BA.5.2	0	0.0%	104	72.7%	NA
BA.5.2.1	0	0.0%	6	4.2%	NA
BA.5.2.2	0	0.0%	8	5.6%	NA
BA.5.3	0	0.0%	1	0.7%	NA
BA.5.3.1	0	0.0%	2	1.4%	NA
BE.1.1	0	0.0%	4	2.8%	NA
C. 36	5	29.4%	0	0.0%	NA
Influenza					
Incidence	7	9.2%	51	11.7%	0.275
Vaccination rate	269	26.8%	1,039	26.9%	0.989
Vaccine breakthrough rate	3/269	1.1%	15/1,039	1.4%	0.906
Subtypes					
A(H1N1)	3	33.3%	10	19.2%	0.431
A(H3N2)	2	22.2%	27	51.9%	0.423
Flu-B	4	44.4%	15	28.8%	0.201
Co-infections					
SARS-CoV-2 and influenza	2	2.6%	1	0.2%	0.036

### Genotyping

#### SARS-CoV-2

Of 143 sequenced SARS-CoV-2 isolates 108 (75.5%) were Omicron variants, 35 (24.5%) probable Omicron. Clades include 58.7% 22B, 5.6% 21 L, 2.1% 21 M, 1.4% BA.5 like, and 7.7% unassigned. Of the 143 isolates, 104 (72.7%) were BA.5.2 lineage, 5.6% BA.5.2.2 and BA.2, 4.2% B.1.1.529 and BA.5.2.1, 0.7% BA.5.3 and BA.5 and BA.2.75 (Table 3).

#### Influenza

Of the 51 patients positive for influenza viruses, 51.9% had A(H3N2) subtype, 28.8% Flu-B and 19.2% had A(H1N1) (Table 3).

### Comparison between Hajj 2022 and Ramadan Umrah surveys

**Fig. 1:**
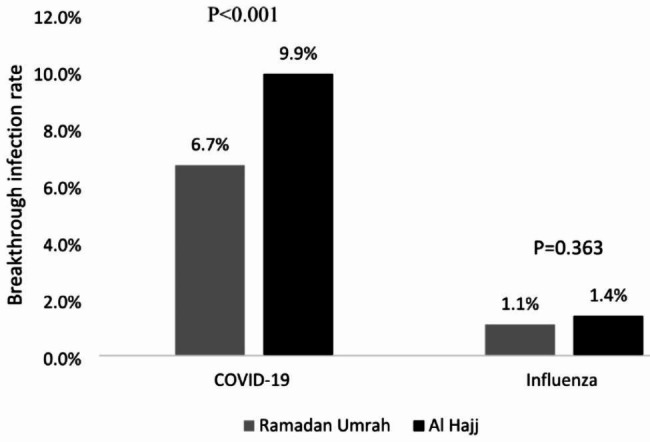
Rate of SARS-CoV-2 and influenza vaccine breakthrough rates among the Egyptian pilgrims returning from Ramadan Umrah compared to Hajj 2022

Egyptian Hajj pilgrims who returned from Hajj 2022 had a higher rate of positivity compared to those who performed Ramadan Umrah in the same year (11.3 vs. 7.6%, *p* < 0.001). Prevalence of COVID-19 and VBTI rates increased significantly among Hajj compared to Umrah pilgrims (9.9 vs. 6.7%, *p* < 0.001) (Table 3; Fig. 1). Influenza prevalence increased insignificantly (11.7 vs. 9.2%, *p* = 0.275), with no increase in influenza vaccination and VBTI rates (26.9 vs. 26.8% and 1.4 vs. 1.1% respectively) between the two surveys (Table 3; Fig. 1). No significant difference was found in influenza subtypes isolated from the Hajj compared to Umrah pilgrims while the percentage of SARS-CoV-2/Flu coinfection decreased significantly among Hajj pilgrims (Table 3).

## Discussion

This study presents the results of a survey conducted during the first Hajj mass gathering held after three years of suspension due to the COVID-19 pandemic. The study estimated SARS-CoV-2 infection rates among Egyptian pilgrims returning from the Hajj accurately since all pilgrims were required to provide a negative RT-PCR test for COVID-19 before leaving for Saudi Arabia.

### SARS-CoV-2

Compared to Ramadan Umrah held in May of the same year, the prevalence of COVID-19 and VBTI rates increased significantly among Egyptian Hajj pilgrims who returned in July in the same year [9]. This could signal a new wave of COVID-19 in the northern hemisphere in 2022 winter enhanced by the rapidly evolving SARS-CoV-2 strains, the change in population behavior, and the waning of immunity to the virus [13]. Reports indicated an increase in the prevalence of COVID-19 in England and Wales recently, suggesting a new wave of the disease is emerging in Europe [14]. Similarly, COVID-19 cases have also increased in many parts of the United States since May 2022, resulting in a double number of hospital admissions [15].

SARS-CoV-2 Omicron variant is evolving and creating new immunity-evading variants; thus, reinfection may occur even for people who have had an Omicron infection in the same year [16]. Recently, researchers reported the emergence of two new Omicron variants namely BA.4 and BA.5 with more transmissibility and resistance to immunity generated by previous variants and most monoclonal antibodies [17]. The highly contagious BA.4 and BA.5 omicron variants are now prevailing in many countries [18–20]. In this study, the most prevalent variant of SARS-CoV-2 was BA.5 and its subvariants, which did not appear before two months among Umrah pilgrims indicating a rapid evolution of the virus.

In spite of the increase in COVID-19 prevalence between the two surveys, it remains lower than other mass gatherings conducted before vaccines were available, indicating that the vaccines remain effective at preventing COVID-19 infection [21]. The study reported no improvement in the vaccine effectiveness after receiving a booster dose of a monovalent vaccine for COVID-19 infection prevention. Many countries initiated the administration of monovalent vaccine booster doses, however, there are concerns about their effectiveness, sustainability, and possible adverse effects [22]. It was suggested that the recent rise in the number of COVID-19 cases could be linked to the slow roll-out of the bivalent vaccine that includes the Omicron BA.4 and BA.5 subvariants in addition to the original strain of SARS-CoV-2 [23]. As such, monovalent vaccines should be viewed as less effective even with booster doses, whereas bivalent vaccines should be considered to improve vaccine effectiveness. Recently the European Union, human medicines committee (CHMP) has recommended authorizing a bivalent vaccine and to further extending the arsenal of available vaccines to protect people against COVID-19 as the pandemic continues and new waves of infections are expected in the upcoming winter season [24]. This recommendation is supported by the findings of our study.

Previous studies suggested that a heterologous mix-and-match vaccination strategy is a promising strategy as it elicits higher antibody titers and broader cross-neutralizing activity against the emerging VOCs than a homologous booster [20]. Contrary to this, the results of the two surveys conducted among Umrah and Hajj pilgrims found no difference in the COVID-19 vaccine breakthrough rate between homologous and heterologous vaccine regimens [9]. The discrepancy may be because the two surveys estimate the effectiveness of heterologous regimens based on real-world data instead of experimental studies with small sample sizes. To improve the effectiveness of the vaccines, researchers recommend post-marketing surveillance with study populations that are larger than those in the trials [25].

There is controversy over the effectiveness of hybrid immunity in preventing COVID-19 infection compared to vaccine-induced immunity. According to a study conducted among healthcare workers in Quebec, hybrid immunity with two to three vaccine doses increased the estimated vaccine effectiveness to 96% for 5 months or more [26]. Similar to a study conducted in Qatar, we found no difference in COVID-19 prevalence between hybrid and vaccine-induced immunity [27]. In all cases, a heterologous vaccination regimen is still an opportunity to increase vaccination programs’ flexibility in response to the fluctuations in supply, especially for countries with scarce vaccine access and when different vaccines are available at different times. This study also did not find a difference in COVID-19 infection prevention between periods since last dose of vaccine administration, perhaps because most pilgrims were vaccinated three months before departure. Additional studies are required to better estimate the effectiveness of different vaccination schedules and regimens.

Study results indicated that the prevalence of COVID-19 was higher among pilgrims who returned early than those who came later. Other cases of SARS-CoV-2 may have been infected and cured during the stay in Saudi Arabia, suggesting a shorter incubation period for the newly evolved Omicron subvariants [28]. In addition, patients with SARS-CoV-2 in this study were less symptomatic and less in need of medical attention, with none of them requiring hospitalization. This may indicate a shift in the epidemiology of SARS-CoV-2 novel subvariants towards mild illness with short incubation, especially in vaccinated individuals [29]. There may be a transition to endemicity at this point.

The study identified symptomatic pilgrims despite having negative RT-PCR results for SARS-CoV-2 and influenza. The list of acute respiratory diseases’ causative agents could include adenovirus, respiratory syncytial virus, Human Metapneumovirus, Human parainfluenza virus, and Norovirus. Expanding the testing panel for patients with AR symptoms is required to monitor the activities of different respiratory viruses.

This study found differences in acute respiratory infection rates among pilgrims residing in different geographic regions similar to those reported in previous studies among the Egyptian population [30]. There are several reasons for this, including varying levels of natural immunity and different levels of prevention and control measures applied in different regions, such as vaccination.

### Influenza viruses

Previous studies suggested that influenza viruses mutate more frequently than Coronaviruses, probably due to a ‘proofreading’ enzyme present in Coronaviruses that correct copying errors. Available sequencing data indicate that SARS-CoV-2 accumulates only two single-letter mutations per month - about half the rate of influenza [31]. This study did not find a significant rise in influenza prevalence or vaccine breakthrough rates between the two surveys in contrast to SARS-COV-2. These findings could raise a question regarding the frequency of influenza virus mutation compared to SARS-CoV-2.

The influenza viruses’ predominant strains in a given setting are identified through a well-established network of more than 150 laboratories in 127 countries around the world constantly monitoring the circulating influenza viruses twice a year. In addition, experts meet to define the predominant influenza viruses in certain places of the world and define the composition of the vaccine, to make sure that the vaccine will really better protect people against the circulating viruses at a given time [32]. An end-to-end integration of influenza and SARS-CoV-2 into all the components and stages of surveillance was recommended by WHO, along with a timely upload of influenza and SARS-CoV-2 genetic sequence data to a public database, so that activity can be monitored, and effective vaccines maintained [33].

Saudi Ministry of Health recommend vaccination against seasonal influenza before international pilgrims arrive in the Kingdom, particularly for those at risk of severe influenza diseases, including pregnant women, children under five years of age, the elderly, and those with underlying health conditions [34]. In addition, Egypt MoHP requires seasonal influenza vaccination for all pilgrims as part of the Saudi visa application process. A seasonal influenza vaccination is available to Egyptian pilgrims at local health offices throughout most of the year, even though it is not always enforced [35]. The influenza vaccination rate among the Egyptian pilgrims returning from Hajj decreased from 98.1% during the 2009 influenza pandemic to 19.7% between 2012 and 2015, whereas influenza prevalence among them increased from 1 to 14% [8, 35]. This study indicated low rates of influenza vaccination with a low prevalence of influenza among Egyptian pilgrims in both surveys. However, this is probably due to the predominance of SARS-CoV-2 which could change when the influenza virus resurges. WHO reported an increase of 35-fold in the number of influenza viruses detected in the temperate zone of the northern hemisphere between 2020 and 2021 [36]. The anticipated resurgence of influenza viruses, as well as the increase in influenza prevalence whenever there is a reluctance to vaccinate observed, clearly demonstrate the importance of influenza vaccination prior to leaving for Hajj [37].

The same influenza subtypes were identified in both surveys indicating that influenza A/H3N2 was predominating with a lower prevalence of Flu-B and trace of H1N1. The same findings were reported by the WHO for the northern hemisphere temperate zone [38]. During season 2022, a study conducted in Russia found that the majority of the A(H3N2) viruses identified belonged to the vaccine strain A/Darwin/9/2021, while influenza B viruses were similar to the influenza B/Austria/1,359,417/2021 vaccine strain recommended by the WHO for season 2022–2023 [39]. This highlights the value of networking in monitoring predominant strains yearly for the vaccine components recommendation.

Although it was expected to see more cases of SARS-CoV-2 and influenza viruses coinfection as influenza resurges, only one case of coinfection was detected among Egyptian pilgrims in July [40]. Recently coinfection with different lineages of the SARS-CoV-2 was reported in different studies rather than coinfection of SARS-CoV-2 and influenza viruses, a finding that was not identified in our study [41, 42]. Monitoring the emergence of new genotypes generated by coinfection and recombination is crucial to detect novel genotypes with unique phenotypic characteristics, including transmissibility and virulence [43].

## Conclusions

COVID-19 prevalence increased among Egyptian pilgrims returning from the Hajj in Saudi Arabia in July 2022, compared to those returning from Umrah in May of the same year, indicating a possible COVID-19 new wave this winter. Vaccine effectiveness for disease prevention was reduced in two months period following the mutation of SARS-CoV-2, while the severity of the disease did not change. There were no differences in COVID-19 prevalence between receivers of different vaccine types, homologous versus heterologous vaccination schedules, hybrid versus vaccine-induced immunity, and full vaccination versus booster dose recipients. Omicron is still the main variant of SARS-CoV-2 with many subvariants mutants identified. A global network could help COVID-19 vaccines be more effective by monitoring predominant SARS-CoV-2 strains.

The study identified a low influenza vaccination and influenza viral infection prevalence rate as well. There were no differences in influenza subtypes or vaccine effectiveness between Hajj and Umrah surveys. Enhancing influenza vaccination is recommended among attendees of mass gatherings including Hajj and Umrah to prevent the resurgence of influenza.

## Data Availability

The datasets generated and/or analyzed during the current study are not publicly available due to privacy restrictions but are available from the Egypt Ministry of Health and Population upon reasonable request.
